# The Effect of Parents’ Literacy Skills and Children’s Preliteracy Skills on the Risk of Dyslexia

**DOI:** 10.1007/s10802-014-9858-9

**Published:** 2014-03-23

**Authors:** Elsje van Bergen, Peter F. de Jong, Ben Maassen, Aryan van der Leij

**Affiliations:** 1 Department of Experimental Psychology, University of Oxford, 9 South Parks Road, OX1 3UD Oxford, UK; 2 Research Institute of Child Development and Education, University of Amsterdam, Nieuwe Prinsengracht 130, 1018 VZ Amsterdam, The Netherlands; 3 Center for Language and Cognition Groningen (CLCG), University of Groningen, Oude Kijk in’t Jatstraat 26, 9712 EK Groningen, The Netherlands

**Keywords:** Dyslexia, Familial risk (FR), Longitudinal, Reading, Arithmetic, Parent–child resemblance, Intergenerational

## Abstract

The combination of investigating child and family characteristics sheds light on the constellation of risk factors that can ultimately lead to dyslexia. This family-risk study examines plausible preschool risk factors and their specificity. Participants (*N* = 196, 42 % girls) included familial risk (FR) children with and without dyslexia in Grade 3 and controls. First, we found impairments in phonological awareness, rapid naming, and letter knowledge in FR kindergartners with later dyslexia, and mild phonological-awareness deficits in FR kindergartners without subsequent dyslexia. These skills were better predictors of reading than arithmetic, except for rapid naming. Second, the literacy environment at home was comparable among groups. Third, having a dyslexic parent and literacy abilities of the non-dyslexic parent related to offspring risk of dyslexia. Parental literacy abilities might be viewed as indicators of offspring’s liability for literacy difficulties, since parents provide offspring with genetic and environmental endowment. We propose an intergenerational multiple deficit model in which both parents confer cognitive risks.

## Introduction

Prospective studies in which children are followed from the preschool years can provide valuable insights into the characteristics of children who go on to develop dyslexia. However, it is still an open question whether the precursors of dyslexia specifically predict dyslexia and reading development or whether they additionally predict dyscalculia and arithmetic development. Furthermore, less is known about the characteristics of the families in which children who go on to develop dyslexia are born. Knowledge about risk factors of dyslexia sheds light on the causal pathways that ultimately lead to dyslexia. These insights might also be useful for tracing children who are at heightened risk of reading failure and need timely support. The current study investigates plausible risk factors for dyslexia present before reading instruction, both at the level of cognitive skills of the child, as well as at the level of family characteristics.

### Risk Factors in Families

Several prospective studies have included children with an increased risk of dyslexia because they are born into families with a history of dyslexia. Depending on the definition of dyslexia, 33 to 66 % of the children at familial risk (FR) have been found to develop dyslexia (Elbro et al. 1998; McBride-Chang et al. 2011; Pennington and Lefly 2001; Scarborough 1990; Snowling et al. 2003; Torppa et al. 2010; van Bergen et al. 2011). The fact that the prevalence of dyslexia is consistently found to be higher in children with a familial history of dyslexia compared to children without such a history is in accordance with a difference between these samples in liability or risk for dyslexia. However, in such a design liability to dyslexia is in fact dichotomized, as children are divided into groups of low and high familial risk (i.e., noFR and FR). Little attention has been devoted to the notion that within the group of FR children those with and without dyslexia probably do not have equal liabilities. Given that dyslexia is influenced by a wide range of environmental and genetic risk factors (Pennington 2006), its underlying liability distribution, however, must be continuously distributed.

In three independent samples (Torppa et al. 2011; van Bergen et al. 2011, 2012) the equal-liability assumption has been tested by comparing the two FR groups on the reading(−related) skills of the parent with dyslexia. Evidence was found against the equal-liability assumption, as the parents of the affected children were more severely dyslexic than those of the unaffected children. Conversely, information regarding possible differences between the groups in reading skills of the spouse, the unaffected parent, is as yet lacking. As a first indication of the effect of both parents, Gilger et al. (1996) found that offspring affection rates were higher in families with two compared to one dyslexic parent (76 % vs. 57 %, respectively). We will present data on parents’ self-reported literacy skills, a valid indicator of tested skills (Snowling et al. 2012). We expected the unaffected parents of the affected children to report more literacy difficulties than those of the unaffected children, mirroring the findings in affected parents.

Differences between the two risk groups in parental reading skills could suggest that these two groups of children differ in genetic predisposition. However, the alternative explanation is that weaker reading parents offer their child a less advantageous literacy environment. Such differences in literacy stimulation might have been the primary cause of children’s differences in later reading success. We will investigate several characteristics of the home literacy environment when children were 3½ years old, and test whether differences are related to children’s reading status 5 years later. The combination of findings on parental literacy skills and home literacy environment sheds light on whether the intergenerational transmission of risks is predominantly via genetic or environmental pathways.

### Risk Factors in Children

Alongside characteristics of families that might indicate heightened risk for dyslexia, we investigated characteristics of kindergartners that could signify increased risk. More specifically, we examined whether differences between FR dyslexia, FR no-dyslexia, and control children are only present after some years of reading instruction, or whether the groups already demonstrate differences before the start of reading instruction. By comparing FR children who go on to develop dyslexia with controls retrospectively, it has been shown that phonological awareness, rapid naming, and letter knowledge are the key precursors of dyslexia. FR studies consistently found that children with dyslexia were impaired across these skills during the preschool years (Elbro et al. 1998; Pennington and Lefly 2001; Scarborough 1990; Snowling et al. 2003; Torppa et al. 2010; van Bergen et al. 2011).

In addition, FR studies offer the interesting possibility to compare the FR children classified as *non*-dyslexic with their peers without FR (controls). Although these FR children do not meet dyslexia criteria, they typically perform less well than controls on reading and spelling tasks after a few years of reading instruction (Boets et al. 2010; Pennington and Lefly 2001; Snowling et al. 2003; but see Torppa et al. 2010 for an exception; van Bergen et al. 2011; van Bergen et al. 2012). These findings provide further support for the continuity of familial risk (Pennington and Lefly 2001; Snowling et al. 2003; van Bergen et al. 2012), and are consistent with a multifactorial model of the aetiology of dyslexia (Pennington 2006).

The somewhat lower literacy skills of the FR children without dyslexia raise the question whether, before the start of reading instruction, they also exhibit mild deficiencies in the cognitive skills underpinning reading, or whether they start off first grade performing as well as controls but experience slower development in reading skills. In previous FR studies the performance of the FR no-dyslexics was equal to or tended to be weaker than controls on phonological awareness, rapid naming, and letter knowledge in kindergarten (Boets et al. 2010; Elbro et al. 1998; Pennington and Lefly 2001; Snowling et al. 2003; Torppa et al. 2010; van Bergen et al. 2011); the only significant difference being for letter knowledge in the study of Elbro and colleagues. However, sample sizes (ranging from 62 to 113) and therefore power was in general moderate. The only large study (Torppa et al. 2010; *N* = 198) did not find significant differences between these two non-dyslexic groups, neither in preliteracy nor later on in literacy skills. Note that the latter is at odds with other studies. The present study has a comparably large sample (*N* = 202) and therefore greater power to detect subtle differences. In an earlier paper with this same sample we reported mild difficulties at the end of Grade 2 of the FR children without dyslexia on literacy and phonological awareness, but not on rapid naming (van Bergen et al. 2012). Accordingly, we expected to find mild difficulties in kindergarten on letter knowledge and phonological awareness, but not on rapid naming.

### Specificity of Precursors

We also examined the specificity of known precursors for dyslexia. Comorbidity rates of dyslexia and dyscalculia are higher than expected by chance (Landerl and Moll 2010). Accordingly, the above mentioned trio of preliteracy skills also could be predictive of arithmetic skills.

A theoretical framework that is useful for studying overlapping and unique underpinnings of reading and arithmetic (dis)ability is Pennington’s (2006) multiple deficit model. The multiple deficit model is built on the multifactorial and probabilistic aetiology of developmental disorders. It postulates that a cognitive developmental disorder is the behavioural outcome of multiple interacting risk and protective factors. Some of these factors influence several disorders (causing comorbidity) and some are specific to one particular disorder. Although the multiple deficit model pertains to disorders like learning disabilities, it may be valid for learning abilities across the range, since learning disabilities are generally viewed as representing the low end of a normally distributed trait. Several findings on reading and arithmetic abilities are in agreement with the multiple deficit model. At the etiological level, both shared and unique genetic effects on reading and arithmetic have been found (Hart et al. 2009; Kovas and Plomin 2007). Also at the cognitive level shared and unique skills have been identified: phonological deficits seem to be specific for dyslexia, magnitude processing deficits seem to be specific for dyscalculia, and rapid naming deficits have found to be present in both disorders (Landerl et al. 2009; van der Sluis et al. 2004; Willburger et al. 2008).

Following Pennington’s framework, the question is whether common precursors of reading and arithmetic fluency affect the processes that are shared between reading and arithmetic or whether they show domain-specific influences. According to De Smedt and colleagues (Boets and De Smedt 2010; De Smedt et al. 2010), reading and arithmetic ability are concurrently associated because word reading and arithmetic fact retrieval both depend upon the quality of phonological representations in long-term memory. If so, we should find a longitudinal relation between phonological awareness and arithmetic. Building on this hypothesis, the speed of retrieval of phonological representations form long-term memory (as tapped by rapid naming) should be related to subsequent arithmetic fluency, since the latter requires quick retrieval of arithmetic facts. Inefficient retrieval of phonologically-coded arithmetic facts leaves limited memory resources for selecting and carrying out appropriate procedures (Hecht et al. 2001). Indeed, cross-sectional studies have shown associations between rapid naming and arithmetic fluency (e.g., Cowan and Powell 2014; van der Sluis et al. 2007). Georgiou et al. (2013) also found that rapid naming, reading, and arithmetic are interrelated. Contrary to the phonological-representations account (Boets and De Smedt 2010; De Smedt et al. 2010) however, regression analyses showed that what rapid naming shares with each of the learning abilities are processing speed and working memory, rather than phonological awareness. In the few longitudinal studies, relations also have been found between phonological awareness and rapid naming at an early age and arithmetic achievement a few years. (de Jong and van der Leij 1999; Hecht et al. 2001). These relationships have not yet been investigated in the context of an FR design.

The current paper will report new data of an on-going longitudinal study (see e.g., van Bergen et al. 2012). Parental literacy and home literacy environment when children were 3½ years old and children’s cognitive skills in kindergarten will be related to their reading and arithmetic skills in Grade 3. Adding to previous FR studies, in the current study we also examined literacy skills of the non-dyslexic parent and the specificity of known precursors of reading. Research questions were 1) Do home literacy environment and the reading skills of both parents relate to offspring risk of dyslexia? and 2) What is the discriminant validity of predictors of later dyslexia? Do they also relate to later arithmetic skills?

## Method

### Participants

Two samples of children of the Dutch Dyslexia Programme (see van der Leij et al. 2013, for an overview) were involved in this study: 132 at high familial risk (FR) and 70 at low familial risk (noFR). Children who had data present at ages 3½ (literacy questionnaire) and/or 6 (kindergarten), as well as at age 9 (third grade) were eligible for inclusion in this study (*N* = 202). The Dutch Dyslexia Programme was approved by the ethical committee. All parents gave written informed consent and all children gave assent for participation.

The FR dyslexia group consisted of 50 dyslexic children at high familial risk for dyslexia. The FR no-dyslexia group comprised 82 children at high familial risk but without dyslexia. Finally, the control group included 64 children at low familial risk and without dyslexia. Six children at low familial risk were categorized as dyslexic and were omitted from group comparisons (because of the small group size) but included in full-sample analyses (to prevent restriction of range in the low-risk sample’s reading ability). All FR children had at least one parent and one close relative with dyslexia. In 13 cases (9.8 %) both parents had dyslexia. Parental dyslexia was always based on reading tests. On average, the scores of the dyslexic parents (i.e., weakest reading parent) belonged to the bottom 5 % on reading fluency. Parents of the noFR children were also tested to confirm they were average to good readers. A detailed description of the assessment of familial risk is given in van Bergen et al. (2012). Assessment of dyslexia in the children was done in Grade 3. Children were considered to have dyslexia when their score on the word-reading fluency task (described below) corresponded to the weakest 10 % in the population (equivalent to a Wechsler score of ≤6.2 - norm scores taken from van den Bos et al. 1994). The Grade 3 assessment took place between January and May. Cut-off scores were adjusted according to the month of assessment. Dyslexia diagnosis was based on reading fluency –rather than accuracy– as is standard practice in Dutch and other transparent orthographies (e.g., de Jong and van der Leij 2003; Wimmer and Schurz 2010). The children with dyslexia also exhibited poor reading accuracy, spelling, phonological awareness, and rapid naming (van Bergen et al. 2012).

Table 1 shows the characteristics of the three groups. Percentages of boys were similar across groups. The FR dyslexic children had lower IQs at 4 years of age than the non-dyslexic children (see van Bergen et al. 2013, for a comprehensive investigation). The groups did not differ in age at the questionnaire administration. The control children were 2 months younger at the kindergarten and Grade 3 sessions, but the groups did not differ in the number of months of reading instruction when seen in third grade. Kindergarten encompasses 2 years in the Netherlands. The kindergarten assessment took place between April and August of the second year, when the children were between 67 and 79 months old. Four children were kept down in Grade 1 or 2. Like the other children, they were seen for the Grade 3 assessment after almost 3 years of reading instruction, when these four children attended Grade 2. Finally, parental education (averaged over both parents; scale 1 (*primary school only*) to 5 (*university degree*)) was lower in the FR groups than in the control group.

**Table 1:** Group characteristics

	Familial risk				
	Dyslexia	No-dyslexia	Control	*N*	*p*
Sample size	50	82	64	196	
No. (%) of boys	30_a_ (60 %)	45_a_ (55 %)	39_a_ (61 %)	196	0.728
Full-scale IQ (at age 4)	105.37_a_ (9.90)	111.08_b_ (10.75)	113.24_b_ (10.53)	193	<0.001
Age in months at assessments					
Questionnaire	41.75_a_ (2.03)	42.52_a_ (3.75)	41.08_a_ (2.73)	81	0.214
Kindergarten	73.02_ab_ (3.31)	73.29_a_ (3.08)	71.85_b_ (3.11)	189	0.023
Grade 3	107.96_a_ (4.65)	107.46_ab_ (3.99)	105.83_b_ (3.97)	196	0.014
No of months reading at assessment					
Grade 3*	26.18_a_ (1.00)	26.23_a_ (0.89)	25.92_a_ (1.12)	196	0.158
Parental education	3.41_a_ (0.72)	3.42_a_ (0.77)	4.16_b_ (0.74)	192	<0.001

The group means are given with standard deviations in parentheses. Numbers or means in the same row that do not share subscripts differ at *p* < 0.05 on the *χ* ^2^-test or Tukey’s test. No. = number; * = 10 months instruction per year

### Measures

When children were 3½ years old, parents were asked to fill out a questionnaire about their own literacy and the home literacy environment. Subsequently, the children were tested on preliteracy skills at the end of kindergarten (at age 6) and on school achievement in Grade 3 (at age 9).

#### Literacy Questionnaire at Age 3½

##### Parental Literacy

Both fathers and mothers were asked about their print exposure and literacy difficulties. Three questions were related to print exposure: How many hours per week do you spend on average on reading for 1) work/study and 2) leisure? and 3) How many hours per week do you spend on average on writing (e-mails, letters, postcards, diary etc.)? Each was scored on a scale ranging from 1 (*less than 1 h a week*) to 5 (*more than 10 h a week*). The print-exposure measure (range 3–15, Chronbach’s α 0.62) was the sum of the scores for these items. Furthermore, three questions (on a scale of 1 to 3) concerned literacy difficulties: 1) Do you think you are a fast, average or slow reader? 2) Do you have trouble following the subtitles on TV? and 3) Do you think you have more, average or less difficulties with spelling than other people? The sum of the scores for these items formed the literacy-difficulties measure (range 3–9, Chronbach’s αs 0.79 and 0.84 for fathers and mothers, respectively). One copy of the questionnaire was sent out per family.

For 78 fathers and 70 mothers we had scores on both the literacy-difficulties measure and reading-fluency tests of words and nonwords, which allowed us to investigate criterion validity. The parents’ reading-fluency tests (see van Bergen et al. 2012, for descriptions) were administered around the time of their child’s birth. The correlation between the literacy-difficulties measure and a composite of word- and nonword-reading fluency was −0.84 for fathers and −0.85 for mothers.

##### Home Literacy Environment

The literacy questionnaire also contained questions regarding the home literacy environment. Both fathers and mothers were asked to indicate the frequency of storybook reading in a typical week on a scale from 1 (*never*) to 5 (*more than five times a week*) and whether they had a magazine or newspaper subscription. Furthermore, parents were asked to estimate the number of books available in the home (1 = *fewer than 20* to 5 = *more than 150*). Finally, part of an existing questionnaire was used to measure cognitive stimulation (Leseman 1994; Sigel 1982) with statements like “I ask my child questions during storybook reading” and “I encourage my child to tell me about what (s)he has done outside or at (pre)school”. There were six statements (Chronbach’s α 0.54), rated on a scale ranging from 1 (*strongly disagree*) to 6 (*strongly agree*).

#### Preliteracy at Age 6

##### Rapid Naming

Serial rapid naming (van den Bos 2003) consisted of 50 randomly ordered patches of colours (black, yellow, red, green, and blue) arranged in five columns of ten symbols each. Before test administration, children practiced by naming the last column. Children were instructed to name the colours column-wise as quickly as possible. The time to completion was transformed to number of colours per second to normalize the score distribution. The split-half reliability for 6-year-olds is 0.80 (van den Bos 2003).

##### Phonological Awareness

Two tests measured phonological awareness: phoneme blending and phoneme segmentation. In phoneme blending (Verhoeven 1993a) the child was required to blend aurally presented phonemes into a word. For example, children listened to the successive phonemes /r/ /u/ /p/ /s/, after which they had to merge this into *rups* [caterpillar]. Phoneme segmentation (Verhoeven 1993b) was the reverse of phoneme-blending. Now the child had to segment a given word into its constituent phonemes. Both phoneme blending and phoneme segmentation began with three practice trials (with feedback). Test items consisted of 20 monosyllabic words per test, increasing from two to five phonemes, with four to six items for each specific number of phonemes. The tests were stopped when all items with the same number of phonemes were failed. Chronbach’s α for both tests is above 0.85 (Verhoeven 2000). In our sample the tests correlated 0.85. A composite score was created by averaging *z*-scores.

##### Letter Knowledge

Both receptive and productive letter knowledge were assessed. The receptive test (Verhoeven 2002) required the child to point from six alternative lowercase letters to the letter that matched a given sound. For instance, the child was asked “Where do you see the/m/of *mooi* (beautiful)?” The knowledge of 32 graphemes (including digraphs) was tested. Chronbach’s α is 0.88 (Eleveld 2005). In the productive knowledge test, children were asked to provide the sound of 34 graphemes (including digraphs), but letter names were also considered correct (Verhoeven 1993a). The randomly ordered graphemes were printed in lowercase in two columns of 17 items each. Chronbach’s α is above 0.85 (Verhoeven 2000). In our sample the tests correlated 0.89. A composite score was created by averaging *z*-scores.

#### School Achievement at Age 9

##### Reading

To assess word-reading fluency, children were given the One-Minute-Test (Brus and Voeten 1972), which consists of a list of 116 words of increasing difficulty. They were asked to read as many words as possible correctly within 1 min. The parallel-forms reliability is 0.90 for Grade 3 (van den Bos et al. 1994).

##### Arithmetic

Two subtests of the arithmetic tempo test (de Vos 1992) were administered: addition and subtraction. Each paper-and-pencil subtest includes 40 problems of increasing difficulty. Per problem two operands have to be added or subtracted (e.g., 13 + 4 = …). All operands and outcomes are below 100. The number of correctly solved problems within 1 min forms the raw score. A total score was computed as the sum of the standard scores over both subtests. Stock et al. (2010) reported a Chronbach’s α of 0.90 and a split-half reliability of 0.93. The correlation between the subtests in our sample was 0.79.

## Results

About half of the participants returned the questionnaire that was sent by mail. However, the percentage of data present was approximately equally distributed among the groups (FR dyslexia: 28/50, 56 %; FR no-dyslexia: 42/82, 51 %, and controls: 32/64, 50 %)^1^. Missing-value analyses showed that the subsamples with and without missing questionnaire data did not differ significantly on parental education, *t*(189.9) = −0.57, *p* = 0.572, word-reading fluency of the weakest-reading parent, *t*(192.2) = −0.17, *p* = 0.865, or word-reading fluency of the child, *t*(193.0) = −1.52, *p* = 0.131. Hence, further analyses of the questionnaire data were deemed appropriate. The preliteracy data were present for 96 % of the children. Grade 3 data were complete, as this was a requirement for inclusion into the study. One outlier on rapid naming in the control group was removed because the score was 3.4 standard deviations above the control group’s mean. Distributions were close to normal, unless stated otherwise.

Differences among the three outcome groups on continuous measures were evaluated using one-way ANOVAs (unless stated otherwise), followed by pairwise comparisons with Tukey’s correction for multiple testing. Group means, one-way ANOVA results, and effects sizes are presented in Tables 2, 3, and 4. Results are described below for group comparisons on family and child characteristics, followed by predictions of children’s reading and arithmetic skills.

**Table 2:** Parental literacy according to children’s literacy outcome

	Familial risk												
	Dyslexia		No-dyslexia		Control						Effect size (Cohen’s *d*)		
Measure	*M*	*SD*	*M*	*SD*	*M*	*SD*	*N*	*F*	*df*	*p*	FRD vs. FRND	FRD vs. C	FRND vs. C
Print exposure													
Father	7.81_a_	3.36	7.53_a_	3.08	9.81_b_	3.06	95	4.99	(2, 92)	0.009	−0.09	0.65	0.75
Mother	7.96_a_	2.79	8.10_a_	3.00	9.03_a_	2.87	98	1.24	(2, 95)	0.293	0.05	0.37	0.32
Dyslexic parent	7.56_a_	2.98	7.08_a_	3.08			66	< 1	(1, 64)	0.532	−0.16		
Non-dyslexic parent	8.23_a_	3.15	8.56_a_	2.84			65	< 1	(1, 63)	0.659	0.11		
Literacy difficulties													
Father	6.68_a_	1.52	6.20_a_	1.83	3.91_b_	0.93	97	29.91	(2, 94)	<0.001	−0.52	−2.98	−2.46
Mother	6.00_a_	1.94	5.10_a_	2.07	3.84_b_	1.17	101	10.83	(2, 98)	<0.001	−0.77	−1.85	−1.08
Dyslexic parent	7.56_a_	0.96	7.24_a_	1.11			66	1.39	(1, 64)	0.244	−0.30		
Non-dyslexic parent	5.19_a_	1.57	4.02_b_	1.31			68	10.88	(1, 66)	0.002	−1.11		

*F*, *df*, and *p*-values refer to an ANOVA for the effect of group. Means in the same row that do not share subscripts differ at *p* < 0.05 on Tukey’s test. Cohen’s *d* is calculated using the *SD*s of the controls

*FRD* familial-risk dyslexia; *FRND* familial-risk no-dyslexia; *C* control

**Table 3:** Preliteracy skills at 6 years and school achievement at 9 years according to literacy outcome

	Familial risk												
	Dyslexia		No-dyslexia		Control						Effect size (Cohen’s *d*)		
Measure	*M*	*SD*	*M*	*SD*	*M*	*SD*	*N*	*F*	*df*	*p*	FRD vs. FRND	FRD vs. C	FRND vs. C
Preliteracy skills (6 years)													
Rapid naming colours	0.58_a_	0.16	0.72_b_	0.18	0.75_b_	0.18	185	14.19	(2, 182)	<0.001	0.79	0.96	0.17
Phonological awareness													
Blending	7.57_a_	5.70	11.56_b_	6.25	14.45_c_	5.14	189	19.51	(2, 186)	<0.001	0.78	1.34	0.56
Segmentation	5.08_a_	4.97	9.46_b_	6.67	12.32_c_	6.09	189	19.49	(2, 186)	<0.001	0.72	1.19	0.47
Letter knowledge													
Receptive	13.61_a_	6.58	21.27_b_	6.59	22.73_b_	7.20	189	27.91	(2, 186)	<0.001	1.06	1.27	0.20
Productive	9.02_a_	6.28	17.66_b_	8.13	18.50_b_	8.22	188	24.89	(2, 185)	<0.001	1.05	1.15	0.10
School achievement (9 years)													
Reading	28.64_a_	8.21	54.72_b_	12.10	61.53_c_	12.24	196	129.98	(2, 193)	<0.001	2.13	2.69	0.56
Arithmetic	−1.35_a_	1.61	0.15_b_	1.79	0.90_c_	1.75	196	24.22	(2, 193)	<0.001	0.86	1.29	0.43

*F* and *df* values refer to an ANOVA for the effect of group. Means in the same row that do not share subscripts differ at *p* < 0.05 on Tukey’s test. Cohen’s *d* is calculated using the *SD*s of the controls

*FRD* familial-risk dyslexia; *FRND* familial-risk no-dyslexia; *C* control

**Table 4:** β-weights and total *R* ^2^ of the multiple regressions predicting school achievement at 9 years from preliteracy skills at 6 years (left hand side) and pooled within-group correlations (right hand side)

	Regression analyses				Correlations			
	Reading		Arithmetic					
Predictor	Without arithmetic	With arithmetic	Without reading	With reading	Arithmetic	Reading	Rapid naming	Phonological awareness
Controlling variable								
Risk	−0.29***	−0.24***	−0.28***	−0.08				
Arithmetic	–	0.25***	–	–	–			
Reading	–	–	–	0.38***	0.45***	–		
Preliteracy skills (6 years)								
Rapid naming colours	0.24***	0.17**	0.28***	0.19**	0.40***	0.48***	–	
Phonological awareness	0.09	0.10	−0.03	−0.06	0.28***	0.46***	0.38***	–
Letter knowledge	0.37***	0.31***	0.26**	0.12	0.39***	0.56***	0.45***	0.71***
Total *R* ^2^	0.51***	0.56***	0.27***	0.34***				

*N* = 190–194. *p < 0.05; **p < 0.01; ***p < 0.001

### Family Characteristics According to Risk and Literacy Status

Parental print exposure and literacy difficulties of the groups can be found in Table 2. The fathers of the control children spent significantly more time on reading and writing than those of the FR children, but maternal print exposure was not to be related to children’s group. These differential patterns could indicate a parent by group interaction. However, in a multivariate analyses (with data of both parents analysed simultaneously) this interaction was not significant, *F*(2, 90) = 1.63, *p* = 0.202, and there was only an effect of group, *F*(2, 90) = 3.73, *p* = 0.028.

To investigate our hypothesis regarding the effects of the dyslexic and the non-dyslexic parent in the FR sample, we subdivided parent couples according to their reading status (dyslexic vs. non-dyslexic^2^). Parental print exposure was unrelated to reading outcome of FR children (see third and fourth line in Table 2). Non-dyslexic parents (*M* = 8.43, *SD* = 2.95) appeared to read and write approximately equally frequent as the parents of control children (*M* = 9.42, *SD* = 2.62), *t*(94) = 1.59, *p* = 0.115.

Concerning literacy difficulties (also in Table 2), parents in the control group reported fewer problems with reading and spelling, as was expected given the selection criteria. The only FR parents that reported similar levels of literacy as the control parents (*M* = 3.88, *SD* = 0.67) were the non-dyslexic parents of the non-dyslexic children (*M* = 4.02, *SD* = 1.31), *t*(71) = 0.59, *p* = 0.560. Interestingly, within the FR sample parental self-reported literacy difficulties (literacy difficulties, for short) seemed to differentiate children with and without dyslexia. This difference was large and significant (Cohen’s *d* = −1.11, *p* = 0.002) for the non-dyslexic parent. We conducted a follow-up 2 × 2 ANOVA on the four means and standard deviations in the bottom left corner of Table 2. The interaction between parental status (dyslexic vs. non-dyslexic) and child outcome (dyslexic vs. non-dyslexic) approached significance, *F*(1, 63) = 3.85, *p* = 0.054.

Regarding home literacy environment, the percentages of fathers/mothers subscribed to a magazine/newspaper were 68 %/68 % in the FR dyslexia group, 73 %/76 % in the FR no-dyslexia group, and 100 %/91 % in the control group. The differences were significant for fathers, suggesting more subscriptions in control families, *χ* ^2^(2, *N* = 101) = 11.86, *p* = 0.003, but not for mothers, *χ* ^2^(2, *N* = 101) = 4.82, *p* = 0.090. A table with detailed descriptive statistics on number of books in the home, shared reading, and cognitive stimulation can be obtained from the first author. Here we only present analytic results in the interest of space. The variable about number of books in the home was strongly skewed and therefore analysed with the nonparametric Kruskal-Wallis test. Overall, the difference between groups was significant, *K*(2, *N* = 102) = 7.01, *p* = 0.030, suggesting more books in the homes of control families (*M* = 4.62, *SD* = 0.75) compared to FR dyslexia (*M* = 4.04, *SD* = 1.14) and FR no-dyslexia (*M* = 4.07, *SD* = 1.11), but pairwise comparisons with adjusted *p*-values were not significant. The frequency of storybook reading was virtually the same over groups for fathers, *F* < 1, and mothers, *F*(2, 97) = 1.09, *p* = 0.340. The only difference was that mothers read more than fathers. Furthermore, parents provided similar levels of cognitive stimulation, *F* < 1.

### Children’s Characteristics According to Risk and Literacy Status

Group means on precursors of reading at the end of kindergarten are shown in Table 3. All group effects on the preliteracy tasks were highly significant (*p*s < 0.001). The FR dyslexia group was slower on rapid naming and knew fewer letters compared to the two non-dyslexic groups, which were statistically indistinguishable. However, the group means on phonological awareness showed a stepwise pattern, with the FR dyslexic children performing lowest, followed by the FR non-dyslexic, and thereafter by the control children. Group differences on preliteracy were not attributable to the group differences on parental education (Table 1), as ANCOVAs with parental education as covariate also yielded highly significant group effects (*p*s < 0.001) and nonsignificant covariate effects (*p*s < 0.288). Controlling for IQ differences (Table 1) in ANCOVAs showed significant effects of IQ (*p*s < 0.001), but all group effects remained highly significant (*p*s < 0.001).

As can be seen in Table 3, groups did not only differ on reading but also on arithmetic ability. When applying a similar criterion for dyscalculia (≤10, or Wechsler scale score ≤6.2, norm scores taken from Melis 2002) as for dyslexia, the percentages of children identified with dyscalculia were found to differ significantly: 42 % (21/50) in the FR dyslexic group, 20 % (16/82) in the FR non-dyslexic group, and 8 % (5/64) in the control group. These differences were confirmed by a chi-square test, *χ* ^2^(2, *N* = 196) = 19.79, *p* < 0.001.

### Prediction of Children’s Reading Skills

After confirming comorbidity, we examined in the full sample how much of the variance in school achievement can be explained by the preliteracy skills, and whether these skills are specifically related to reading or arithmetic. To ensure that we could collapse the FR and noFR samples, we checked whether the relations between preliteracy skills and school achievement were similar in the two samples. Therefore, we ran regressions with reading or arithmetic as dependent variable, entered one of the preliteracy skills and risk status (coded as 0 = *noFR*; 1 = *FR*) in the first step, and checked in the second step whether the interaction between the latter two explained additional variance. None of the interactions was significant (0.172 ≤ *p*’s. ≤ 923), indicating similar relations in the two samples. Therefore, it was sufficient to include only the main effect of risk in the regression analyses presented below.

The pooled within-group correlations between preliteracy and school skills and results of multiple regression analyses are presented in Table 4. Correlations among all variables were significant. The preliteracy skills were related to both arithmetic and reading skills, albeit seemingly more so with reading.

The first multiple regression showed that risk status and preliteracy skills together explained 51 % of the variance in reading; letter knowledge (akin to an autoregressor) made the strongest contribution. Phonological awareness did not explain variance in reading above that accounted for by rapid naming and letter knowledge. However, when letter knowledge (which correlated 0.71 with phonological awareness) was excluded, rapid naming (β = 0.32, *t*[185] = 5.36, *p* < 0.001) as well as phonological awareness (β = 0.33, t[185] = 5.41, *p* < 0.001) were significant.

When risk and arithmetic ability were added to the model (Table 4, second column), rapid naming and letter knowledge remained significant predictors, so they explained unique variance. This confirms the specific relation between preliteracy and literacy skills. With respect to arithmetic, risk and preliteracy skills explained 27 % of the variance. Rapid naming made a significant unique contribution to arithmetic after risk and reading were controlled.

Lastly, we examined the utility of parental literacy difficulties in predicting children’s reading skills. Again, we first checked the interactions between predictors and risk status in separate regressions, but they were not significant (0.172 ≤ *p*’s. ≤ 911). Hence, only risk status was accounted for in the regressions below.

Three hierarchical regression models were specified (see Table 5). In line with our research question regarding the non-dyslexic parents of the FR children, we again subdivided parents according to reading ability. The weakest-reading parent refers to the dyslexic parents of the FR children and the weakest of the parent couple of the noFR children (although still reading at least average).^3^

**Table 5:** Hierarchical regression models predicting children’s reading at 9 years from parental literacy difficulties

Model	Step	Predictor	Δ*R* ^2^	β^a^
1 (*N* = 98)	1	Risk	0.11***	−0.10
	2	Literacy of weakest-reading parent	0.02	−0.17
	3	Literacy of best-reading parent	0.11***	−0.34***
2 (*N* = 98)	1	Risk	0.11***	−0.10
	2	Parental education	0.03	0.06
	3	Literacy of weakest-reading parent	0.01	−0.16
	4	Literacy of best-reading parent	0.09**	−0.32**
3 (*N* = 86)	1	Risk	0.14***	−0.04
	2	Parental education	0.43***	0.15
		Children’s preliteracy skills		
		Rapid naming		0.28**
		Phonological awareness		0.14
		Letter knowledge		0.34**
	3	Literacy of weakest-reading parent	0.01	−0.19
	4	Literacy of best-reading parent	<0.01	−0.06

^a^Reported β’s are values at the final step (all predictors included)

*p < 0.05; **p < 0.01; ***p < 0.001

Risk status was mainly based on the weakest-reading parent. For that reason we anticipated that knowledge of the self-reported literacy difficulties of the weakest parent would not add much over and above risk status. Therefore, in subsequent analyses literacy difficulties of the weakest-reading parent were entered before those of the best-reading parent. This allowed us to test whether literacy difficulties of the best-reading parent (which were not crucial in sample selection) are related to their offspring’s reading skills, controlling for literacy difficulties of the weakest-reading parent. It appeared that literacy difficulties of the weakest-reading parent indeed did not explain a significant amount of variance in children’s reading fluency, but differences in literacy difficulties of the best-reading parent explained an additional 11 % of the variance. Over and above risk, parental education, and literacy difficulties of the weakest-reading parent, the literacy difficulties of the non-dyslexic parent accounted for an additional 9 % in children’s reading fluency. In a final regression analysis, differences in risk, parental education, and children’s preliteracy skills together explained an impressive 57 % of the variance in children’s reading fluency, yet parental literacy difficulties did not significantly add to this prediction.

The regression analyses show the impact of the best-reading parent on children’s reading outcome. Within the FR sample this pertains to the effect of the non-dyslexic parent on their offspring’s risk for dyslexia. To illustrate this effect, we dichotomised the literacy-difficulty measure: ‘non-dyslexic’ parents scoring ≥6 4 were categorized as having literacy difficulty. It appeared that within the group of FR children with a non-dyslexic parent *without* literacy difficulties 30 % (16/54) developed dyslexia, compared to 79 % (11/14) in the group of FR children with a ‘non-dyslexic’ parent *with* literacy difficulties.

## Discussion

The current paper studied putative risk factors for dyslexia at the level of literacy behaviours and skills of parents, and at the level of cognitive skills of kindergartners. This was done utilizing data of an ongoing longitudinal study in which children with and without familial risk are followed from infancy.

### Intergenerational Transfer

An important issue addressed was the intergenerational transfer of reading skills. We measured parental reading and spelling difficulties using a rating scale (assessed when children were 3½), which demonstrated high validity: self-reported and tested reading performance in a subsample correlated 0.84-0.85. First, we investigated within the FR sample the effect of the non-dyslexic parent, whose contribution has been neglected in previous work. We found that self-reported literacy difficulties (i.e., reading and spelling difficulties) of the non-dyslexic parent differentiated FR children with and without dyslexia, that is, those of affected children reported on average more difficulties themselves. This supports the view that children who go on to develop dyslexia have a higher genetic predisposition towards dyslexia and that parental skills are indicative of their children’s liability (van Bergen et al. 2012). Second, the literacy difficulties of the dyslexic parent, conversely, did not differentiate FR children with and without dyslexia and did not explain variance in children’s reading. Put differently, their self-reported difficulties did not add to the prediction of children’s reading beyond the fact that they have dyslexia. However, in an earlier paper about this sample (van Bergen et al. 2012) we showed that individual differences in an objective measure of parental word-reading fluency did differentiate FR children with and without dyslexia and did explain variance in children’s word-reading fluency. The group difference on this objective measure was somewhat larger than on the self-reported measure (Cohen’s *d* = 0.48 vs. 0.30, respectively), presumably reflecting that measuring skills is still more reliable than asking about skills (Snowling et al. 2012). Third, intergenerational transfer of literacy skills was also observed in the FR and noFR samples combined: in regression analyses parental literacy difficulties predicted children’s reading fluency, even after accounting for risk and differences in educational level of both parents. When additionally children’s preliteracy skills were taken into account, parental literacy was no longer predictive.

Only recently an interest has emerged in the predictive value of parental skills for the development of children’s reading skills. Previous FR studies have found associations between reading accuracy, reading fluency, spelling, phonological skills, and vocabulary of the dyslexic parent and their offspring’s reading outcome (Torppa et al. 2011; van Bergen et al. 2011, 2012). Most importantly, this is the first familial-risk study that demonstrates the importance of the literacy skills of the parent without dyslexia. They predicted children’s reading beyond the effect of the dyslexic parent. Within the children with a dyslexic parent, the risk for dyslexia was 2½ times higher if the other parent had literacy difficulties as well. Although the sample size was too small for a reliable estimate of the relative risk, it clearly demonstrates the elevated risk of having a second parent with literacy difficulties (in line with Gilger et al. 1996).

### Home Literacy Environment

Investigation of environmental influences did not indicate links between children’s home literacy environment and children’s reading outcome. Before school entry, the home environment of FR children with and without dyslexia did not differ in terms of shared reading, cognitive stimulation, how much parents read and write, newspaper subscriptions, and number of books, although control families tended to have more newspapers and books. Likewise, in previous FR studies no group differences have been found in shared reading, neither in children’s access to print, like library membership or number of books at home (Elbro et al. 1998; Torppa et al. 2007; van Bergen et al. 2011). Although many educators and parents have strong beliefs about the impact of story book reading on later reading success, research repeatedly fails to find such an association (see for a review Sénéchal and Young 2008), which is in line with the present findings.

The absence of a relation between preschool home literacy environment and subsequent reading attainment found in the current and previous familial-risk studies is in agreement with behavioural genetic studies showing low levels of shared environment influence, whereas up to 80 % is due to genetic influences (e.g., Byrne et al. 2009; Haworth et al. 2009). However, the possibility remains that environmental influences in FR studies are downplayed because they are not measured thoroughly, and because FR studies are based on voluntary samples and therefore might include a somewhat restricted range of environments. Another issue that challenges studying environmental effects is that if effects of environmental experiences are found at all, it might well be that those experiences are partly under genetic control (see e.g., Kendler and Baker 2007). Put differently, there may well be forms of gene-environment correlations at play, not just main effects. As an example of an active gene-environment correlation, results of Scarborough et al. (1991) suggested that, compared to pre-schoolers who did not become dyslexic, future dyslexic pre-schoolers were less read to because they were less interested in books. So children’s early language and cognitive development already seems to influence the amount of literacy-related activities they seek.

### Preliteracy Skills

In line with the literature, in kindergarten the children who went on to develop dyslexia were impaired on rapid naming, phonological awareness, and letter knowledge. Interestingly, the FR children without later dyslexia had age-adequate rapid naming and letter knowledge, but were mildly impaired on phonological awareness. Despite adequate letter knowledge and rapid naming before the start of formal reading, these children read less fluently in Grade 2 (van Bergen et al. 2012) and Grade 3 (Table 3). On the other hand, their reading scores fell within the normal range despite their family history. Hence, it might also be argued that good letter knowledge and skills tapped by rapid naming appear to act as protective factors for dyslexia.

The different results for letter knowledge and phonological awareness were unexpected given the often observed reciprocal development between these skills (e.g., de Jong 2007; Wagner et al. 1994). However, teaching of letters by parents affects children’s letter knowledge (Torppa et al. 2006). The dyslexic families in our study are possibly aware of their children’s risk for dyslexia and might have paid extra attention to teaching letters, which might explain the good letter knowledge of the FR no-dyslexia children. This account is in line with the higher treatment fidelity found in FR families during intervention in kindergarten (Zijlstra et al., The prevention of dyslexia in children with and without familial risk: A randomized controlled trial, submitted). Alternatively, the learning capacity of these children is less affected and more similar to that of control children, making them more likely to pick up letter-sound knowledge.

The finding of weak phonological awareness in combination with good rapid naming of the FR no-dyslexics in kindergarten mirrors the pattern found in this group at the end of second grade (van Bergen et al. 2012), indicating longitudinal stability. Poor performance on phonological awareness has been hypothesized (e.g., Boets et al. 2011; Goswami 2011; Tallal 1980) to be a developmental consequence of impairments in basic auditory processing. Basic auditory processing has been studied in a subset of the current sample at the age of 1½ (van Zuijen et al. 2012) and 3½ (Plakas et al. 2013) using event-related potentials and was indeed found to be weak in FR compared to noFR children. However, basic auditory processing was more strongly related to later reading fluency than phonological awareness, questioning a causal chain from basic auditory processing to reading fluency via phonological skills.

The different group patterns observed for phonological awareness and rapid naming highlight that they have partially unique influences on reading ability and disability, as proposed by the double-deficit theory (Wolf and Bowers 1999). Although their correlation with later reading was remarkably similar (~0.45), group differences showed different patterns for the two precursors and the regression analyses revealed partly unique contributions to reading. In line with this, van den Boer et al. (2013) recently demonstrated differential effects of phonological awareness and rapid naming on the developing reading system. In an experiment with words of different lengths, they modeled the amount of serial (or letter-by-letter) processing as indexed by the time needed to read each additional letter (the word-length effect). They disentangled the word-length effect from overall reading speed and showed that phonological awareness is associated with the degree of serial processing, whereas rapid naming is related to overall reading speed, irrespective of the degree of serial processing. Van den Boer et al. argue that “poor phonological awareness results in poor buildup of orthographic representations, and therefore in continued reliance on a serial processing strategy” (p.244/245). Translating their conclusions to the current study, the FR no-dyslexia group is hypothesized to have some difficulty with building up orthographic knowledge (a requisite for sight-word reading), but not with fast processing of orthographic knowledge and fast retrieval of phonological codes. The FR dyslexia group seems to be impaired in all these elements.

### Specificity of Preliteracy Skills

Our study once more confirmed that poor rapid naming, phonological awareness, and letter knowledge in kindergarten are cognitive risk factors for dyslexia. With regard to arithmetic, our study showed a clear stepwise pattern of (co)morbidity with dyscalculia. Rates of dyscalculia in the FR dyslexia, FR no-dyslexia, and control groups were 42 %, 20 %, and 8 %, respectively. The demonstrated association between reading (dyslexia) and arithmetic (dyscalculia) raises the question as to whether the three cognitive risk factors for dyslexia are specific for reading. Our analysis controlling for risk and arithmetic showed that rapid naming and letter knowledge or phonological awareness uniquely captured variance in later reading skills. Vice versa, only rapid naming had a small but significant influence on arithmetic, while holding reading constant (in agreement with de Jong and van der Leij 1999), despite the clear associations between the three preliteracy skills and subsequent arithmetic achievement. These results suggest that the preliteracy trio is rather specifically related to the academic domain of reading (see for similar findings from kindergarten to Grade 1 Georgiou et al. 2013). Note that our measurement methods of reading (word-decoding fluency) and arithmetic (arithmetic fluency) closely resemble each other. As lower overlap between word reading and broader mathematical skills are to be expected, this strengthens the finding that letter knowledge and phonological awareness are stronger predictors of reading. In a recent study (Koponen et al. 2013) similar specificity was found for phonological awareness as a reading-specific precursor, whereas rapid naming was as strongly related to later arithmetic fluency as to later reading fluency. Together, our studies suggest that rapid naming should not be considered as a domain-specific precursor.

In our data, the part of the variance of letter knowledge and phonological awareness that was predictive of later arithmetic performance was shared with reading performance, as indicated by the lack of unique contributions over and above reading. This is in line with Hecht et al. (2001) who also did not find a unique effect of phonological awareness on arithmetic over a 3-year time period, after controlling for individual differences in reading ability. Fluent word-level reading and arithmetic fact retrieval have in common that they are both affected by the quality of phonological representations (Boets and De Smedt 2010; De Smedt et al. 2010) and include verbal learning. Note that this hypothesis, that explains why reading and arithmetic are associated, is not directly testable with the current arithmetic task with items of increasing difficulty. Children likely started the task using fact retrieval and shifted somewhere along to using procedures. Nevertheless, fact retrieval is likely an element in the more difficult calculation as well, because procedural strategies often involve the (partly) breaking down of complex calculations into simple ones that can be solved by fact retrieval. Based on the findings of De Smedt and colleagues higher correlations with arithmetic might be expected when arithmetic was tested using only items with a high probability of being solved purely by retrieval.

Rapid naming, interestingly, uniquely predicted arithmetic while holding reading constant, and vice versa. The importance of efficient retrieval of phonological codes (as tapped by rapid naming) for arithmetic efficiency has also been shown in cross-sectional work (e.g., van der Sluis et al. 2007) and supports the view that efficient retrieval of arithmetic facts leaves sufficient memory resources for the selection and implementation of appropriate procedures (Hecht et al. 2001). Contrasting with de Smedt’s phonological explanation, but in line with the findings of Georgiou et al. (2013) it is not the phonological but the processing-speed component of rapid naming that explains its relation with arithmetic. Our study differs however from Georgiou et al.’s study in terms of arithmetic task, age span and sample selection, hampering drawing parallels. Data of a more similar study (de Jong and van der Leij 1999) showed specific effects of rapid naming on reading from kindergarten to Grade 2, and a trend for a specific effect on arithmetic (see their Table 9), mirroring the current findings from kindergarten to Grade 3. The conclusion that the aspects of rapid naming that impact upon reading and arithmetic development only partly overlap is intriguing and calls for further research to uncover which cognitive aspect of rapid naming is exclusively related to arithmetic fluency.

It should be acknowledged that we had a specific sample of children with and without FR for dyslexia. However note that the relations among variables were similar in the FR and noFR samples, providing some support for the generalizability of the current results to other populations. The finding of equally stimulating home environments among groups is weakened by the limited reliability of the questionnaire. However, the finding fits with the body of family and twin studies. Regarding comorbidity, the current study underlines the value of studying simultaneously the emergence of two developmental disorders. Inclusion of other disorders that often co-occur with dyslexia in future longitudinal studies will further contribute to our understanding of dyslexia.

### Intergenerational Multiple Deficit Model

Our findings fit well in Pennington’s multiple deficit model (2006). Deficits in phonological awareness and rapid naming can be viewed as two of the multiple cognitive deficits that increase the probability of dyslexia. Poor letter knowledge can be seen as a third underlying cognitive deficit, or as an earlier developmental manifestation of dyslexia (akin to an autoregressor of reading). Slow rapid naming might well be a cognitive deficit in common to dyslexia and dyscalculia (in line with e.g., van der Sluis et al. 2004; Willburger et al. 2008). Shared deficits can account for the frequent co-occurrence of these developmental disorders. Rapid naming taps multiple cognitive processes and it is yet to be scrutinized which of its components affect both the developing reading and arithmetic system and which are unique to each of these domains.

The parent–child resemblance reported in the current and other recent literature seems to imply that the multiple cognitive deficit model of Pennington (2006) can be extended to an intergenerational multiple cognitive deficit model (see van Bergen et al., The intergenerational multiple deficit model and the case of dyslexia, in revision, for a more elaborate justification and description). As we argued in an earlier paper (van Bergen et al. 2012), literacy abilities of parents might be viewed as indicators of their offspring’s risk or liability for literacy difficulties, since parents provide their offspring with their genetic and family endowment. In Pennington’s model the focus is on a specific individual, whereas we propose to add an extra layer, or level of analysis which encompasses parental characteristics. In the intergenerational multifactorial deficit model, characteristics of both parents can be seen as a proxy for the aetiological risk and protective factors for their offspring’s predisposition towards developmental disorders. These parental characteristics include both factors that directly shape children’s environmental exposure, and cognitive factors that are partly genetically transmitted to their offspring. The latter may include (when children’s reading is the outcome of interest) skills like reading and spelling, or may include their cognitive underpinnings like phonological awareness, rapid-naming ability and verbal short-term memory. Examples of parental characteristics that might affect children’s outcome via direct environmental influences are, in the case of reading development, the frequency of shared reading (fostering print knowledge and interest) and the frequency of independent reading (providing a role model). On each of these cognitive and environmental continua parents occupy a position in multivariate space. The constellation of these factors of both parents gives an indication of their offspring’s liability to develop certain cognitive disorders. The findings from the Dutch Dyslexia Programme suggest that parents confer risk factors for dyslexia predominantly via genetic rather than environmental pathways. That is, genetic transmission and passive gene-environment correlations might be more important than direct environmental effects of parental characteristics.

More research investigating the cognitive risks that parents pass on to their children is needed to further develop the proposed intergenerational multiple deficit model. Such studies will help to contribute to unravelling the aetiology of developmental disorders. Additionally, such research could lead to better identifying the children at-risk for cognitive developmental disorders and provide them timely with appropriate support.

## Footnotes

1 Regarding self-reported literacy difficulties, for 98 families both parents had filled out those questions and for 4 families (2 FR dyslexia and 2 FR no-dyslexia) only the mother.

2 In 9 out of the 70 FR children with questionnaire data present, both parents had dyslexia. ‘Dyslexic parent’ refers to the weakest reading parent and ‘non-dyslexic’ to the other parent.

3 The literacy variables in the noFR group showed strong floor effects indicating few to no difficulties. Nevertheless, in line with insignificant risk-status X predictor interactions, analyses in just the FR sample yielded the same pattern findings as those presented below for the entire sample.

4 This cut-off score was chosen because parents scoring ≥6 indicated to have difficulties on at least one of the three questions. Moreover, the score distribution showed a jump between score 5 and 6.
